# Opioid Misuse and Asthma and Allergic Diseases in College Students: The Role of Race and Ethnicity

**DOI:** 10.1016/j.focus.2026.100525

**Published:** 2026-07-07

**Authors:** Fares Qeadan, Stephane Beaudin, Aisha Arshad, William A. Barbeau

**Affiliations:** Parkinson School of Health Sciences and Public Health, Loyola University Chicago, Maywood, Illinois

**Keywords:** Opioid misuse, asthma, allergies, college students, race ethnicity

## Abstract

**Introduction:**

Opioid misuse, asthma, and allergies are major drivers of morbidity in the U.S. and are disproportionately distributed by race/ethnicity. Emerging evidence suggests that opioid misuse may be linked to an increased risk of asthma and allergic diseases. This study estimates the prevalence and association of opioid misuse, asthma, and allergies in a national sample of U.S. college students and assesses whether race/ethnicity, gender, and age modify this association.

**Methods:**

A cross-sectional study using data from the 2019–2024 American College Health Association’s National College Health Assessment IIIb was conducted. The prevalence of self-reported diagnoses of asthma, environmental allergies, food allergies, and pet/animal allergies by self-reported opioid misuse was estimated. Multivariable logistic regression was used to estimate the AORs. Interaction terms between opioid misuse and race/ethnicity, gender, and age were estimated to test for effect modification.

**Results:**

In a sample of 535,909 college students, the prevalence of asthma, environmental allergies, food allergies, and pet/animal allergies was 21.5%, 34.6%, 14.8%, and 17.1%, respectively, among students who reported opioid misuse. Race/ethnicity was a significant effect modifier of the association between opioid misuse and asthma (*p*=0.0329), food allergies (*p*=0.0032), and pet/animal allergies (*p*<0.0001). Opioid misuse was generally associated with higher odds of asthma and allergic outcomes, with particularly strong associations for asthma, food allergies, and pet/animal allergies in selected racial/ethnic groups. Among non-Hispanic Asian or Asian American students, the AORs were 1.47 (95% CI=1.23, 1.74) for asthma, 1.53 (95% CI=1.28, 1.82) for food allergies, and 1.71 (95% CI=1.44, 2.03) for pet/animal allergies.

**Conclusions:**

This study provides additional epidemiologic evidence of a link between opioid misuse and asthma and allergic diseases in a large sample of mostly young adult college students. Evidence of effect modification by race/ethnicity highlights potential social and environmental exposures that contribute to the link between opioids and asthma/allergies.

## HIGHLIGHTS

• Opioid misuse was reported by 3.8% of college students.
• Asthma and allergy diagnoses were more common with opioid misuse.
• Race/ethnicity modified asthma, food allergy, and pet allergy associations.
• Asian and other/missing groups showed elevated associations.
• Findings highlight social and environmental contexts of risk.

## INTRODUCTION

The opioid crisis, a challenging public health emergency in the U.S., has disproportionately affected young adults, manifesting as widespread and escalating misuse of opioids. This crisis, characterized by an alarming rise in addiction, overdose incidents, and mortality, presents a complex challenge to both public health and healthcare systems.^1^^,^^2^ Opioids, although crucial in pain management, harbor a high potential for misuse, leading to myriad health and social complications.^3^ Young adults, those aged 18–25 years, are disproportionately impacted by the opioid crisis, as evidenced by the 2021 National Survey on Drug Use and Health, which reported that this demographic accounts for approximately 1 million of the 8.7 million adults misusing opioids in the past year.^4^^,^^5^ This age group’s vulnerability to the addictive properties of opioids often results in adverse health outcomes.^6^^,^^7^ The 2020 findings from the U.S. national Monitoring the Future follow-up study concerning substance use among college students and young adults further underscore this concern. Although lifetime prevalence estimates of heroin or other narcotic use have decreased over the past 10 years, in 2020, the lifetime prevalence among college students was 0.4% for heroin use and 4.1% for other narcotic use.^8^ The staggering number of opioid-related overdoses, with an age-adjusted rate of 24.0 per 100,000 for any opioid-related overdose deaths in 2023, highlights the gravity of this crisis.^9^ Specifically, among college students, the prevalence of opioid misuse fluctuated between 4.0% and 7.0% in Spring 2019, among different race/ethnicity groups.^10^^,^^11^

Concurrently, the potential impact of opioid misuse on allergic diseases and asthma, especially among young adults and college students, has emerged as a critical area of concern. Asthma, a chronic respiratory disorder, and allergies, characterized by abnormal immune responses, significantly affect this demographic. The prevalence of current asthma in U.S. adults in 2022 was 8.7%.^12^ In addition, about 25.7% of adults in 2021 had a seasonal allergy, 7.3% had eczema, and 6.2% had a food allergy.^13^ Among college students, the American College Health Association (ACHA) reported that 12.2%, 13.3%, and 29.7% of students, in Fall 2021, reported ever being diagnosed with food, animals/pets, and environmental allergies, respectively, and estimated a 15.7% prevalence of lifetime asthma.^14^ Factors such as air pollution, antibiotics, obesity, and genetic predispositions further exacerbate the risk of these diseases.^15^ The health, economic, and quality-of-life impacts of these conditions are profound. A national analysis estimated the annual U.S. cost of asthma, including medical costs, absenteeism, and mortality, at $81.9 billion in 2013.^16^ Asthma also imposes direct and indirect costs among school-age children through medical expenditures, school absenteeism, and parental productivity losses.^17^ Annual medical expenses associated with pollen allergies surpass $3 billion, with prescription medications accounting for almost half of these costs.^18^ The global trend mirrors this rise, necessitating a comprehensive investigation into the myriad risk factors associated with these illnesses.^19^, ^20^, ^21^

Despite the concurrent challenges of opioid misuse and allergic diseases, research exploring their direct association remains scarce,^22^ especially among college students. The current vast literature primarily focuses on the central nervous system and the addictive effects of opioids, with limited exploration of their impact on immune function and allergic conditions.^23^ This gap is significant, considering that preliminary studies hint at a potential link between opioid-induced immunodeficiency and the immunologic pathophysiological processes involved in allergic disorders.^24^, ^25^, ^26^ Proposed mechanisms that may link opioid exposure to the pathophysiology of asthma and allergic conditions include immunomodulatory effects on innate and adaptive immune function and opioid-triggered mast cell activation with histamine release, which can produce allergy-like symptoms and may plausibly exacerbate respiratory symptomatology.^26^, ^27^, ^28^, ^29^ In addition, the endogenous opioid system is involved in the itch response, with μ-opioid receptor activation promoting itch and κ-opioid receptor activation inhibiting itch.^30^ Naltrexone, a μ-opioid receptor antagonist, has shown promise as a treatment for pruritus associated with various conditions such as systemic sclerosis and atopic dermatitis.^31^, ^32^, ^33^ At the same time, the relationship may be bidirectional, such that individuals with asthma or allergic disease may be more likely to receive opioid prescriptions for pain or have comorbid mental health conditions associated with misuse. Shared social/behavioral risk factors may also confound observed associations. This is supported by epidemiologic evidence, because a matched retrospective cohort study with over 2 million patients found that those with asthma were 2.12 times more likely to develop incident opioid use disorder than patients without asthma.^22^

Moreover, the role of potential effect modifiers, such as race and ethnicity, age, and gender, in this relationship is underexplored, despite indications that ethnic minorities may disproportionately suffer, independently, from both the opioid crisis and the prevalence of allergic diseases.^11^^,^^34^^,^^35^ Race and ethnicity are social constructs that often index differential exposure to structural and environmental conditions (e.g., residential segregation, neighborhood socioeconomic position, pollution, housing quality, and access to preventive care) that shape the risk of asthma and allergic disease as well as differential exposure to the opioid crisis. Thus, the hypothesis was that the relationship between opioid use and asthma and allergic diseases varies across race and ethnicity, not owing to inherent biological differences but owing to differential exposure to social and environmental determinants of opioid use and asthma and allergic disease.^36^, ^37^, ^38^

To address this research gap, this cross-sectional study used data from the ACHA’s National College Health Assessment (NCHA) IIIb from 2019 to 2024 to examine the association between opioid misuse and asthma/allergies among college students, with a particular focus on the potential modifying effects of race/ethnicity, gender, and age.

## METHODS

### Study Sample

This study used the ACHA-NCHA IIIb survey data from 2019 to 2024. The ACHA-NCHA IIIb is a nationally distributed biannual survey that collects student health and health-related behavior data from 830 participating postsecondary educational institutions, with participating institutions varying by academic semester. The ACHA-NCHA IIIb survey inquires about physical and mental health, safety, substance use, health behaviors, and demographic characteristics.^39^ Participating institutions either send surveys to the entire student body or to a random sample of the student body. Response rates varied by semester from 10% (Fall 2023) to 14.1% (Spring 2020).^40^ The ACHA-NCHA IIIb data from Fall 2019 through Spring 2024 included a total of 541,093 students. Students with missing responses to the primary exposure of this study, opioid misuse, were excluded, yielding a final analytic cohort with a sample size of 535,909. This study was reviewed and determined to be exempt from IRB oversight by Loyola University Chicago (LU# 219803) under 45 CFR 46.104(d)(4)(i) as secondary research using deidentified data.

### Measures

The 4 primary outcomes of interest were self-reported diagnoses of asthma, environmental allergies, food allergies, and pet or animal allergies. Students were asked, *Have you ever been diagnosed by a healthcare or mental healthcare provider with any of the following ongoing or chronic conditions?* with *Asthma, Allergies - environmental (for example: pollen, grass, dust, mold), Allergies - food allergy*, and *Allergies - animals/pets* listed as conditions. For each ongoing or chronic condition, students could either respond *No* or *Yes*.

The primary exposure of interest was self-reported opioid misuse. Students were asked, *In your life, which of the following substances have you ever used? For prescription medications, please report nonmedical use only. “Nonmedical use” means taking prescription drugs just for the feeling or experience they cause or taking them more often or at higher doses than prescribed*. This question presented survey participants with a list of substances, with yes/no as possible selections for each substance. Included in the list of substances were *Heroin* and *Prescription opioids (morphine, codeine, fentanyl, oxycodone [OxyContin, Percocet], hydrocodone [Vicodin], methadone, buprenorphine [Suboxone], etc.) [Please report nonmedical use only].* Opioid misuse was dichotomized as yes if they reported ever using heroin or the nonmedical use of prescription opioids and as no otherwise.

Race/ethnicity was considered a potential effect modifier and was assessed by the question, *How do you usually describe yourself? (Please select ALL that apply).* Possible responses included *American Indian or Native Alaskan, Asian or Asian American, Black or African American, Hispanic or Latino/a/x, Middle Eastern/North African (MENA) or Arab Origin, Native Hawaiian or Other Pacific Islander Native, White, Biracial or Multiracial*, and *My identity is not listed above*. If participants marked American Indian or Native Alaskan (AI/NA) alone or in combination with any other identity, then they were coded as AI/NA. If participants marked Hispanic or Latino/a/x alone or in combination with any other identity (except AI/NA), then they were coded as Hispanic or Latino/a/x. If participants marked 2 or more identities or biracial or multiracial, then they were coded as non-Hispanic (NH) biracial or multiracial. If participants marked only 1 option—Middle Eastern/North African (MENA) or Arab Origin, Native Hawaiian or Other Pacific Islander Native, My identity is not listed, or did not endorse any of the listed options—then they were coded as NH other/missing. Otherwise, participants who marked only 1 option were coded as the identity they marked.

Gender identity and age were also considered as potential effect modifiers. Gender identity was constructed from 3 questions: (1) *What sex were you assigned at birth?*, (2) *Do you identify as transgender*, and (3) *Which term do you use to describe your gender identity*? Students who endorsed a transgender identity in either question were categorized as transgender in this study. Otherwise, students who endorsed any identity or sex other than man or male or woman or female were categorized as gender diverse. Otherwise, students were categorized as cisgender male or cisgender female according to their responses. Age was assessed by asking, *how old are you?* Age was categorized to allow for a nonlinear relationship between age and the log odds of the outcomes. Age was categorized into 18–19, 20–22, 23–25, 26–30, >30 years on the basis of the distribution and percentiles of age in the overall sample.

Health behavior–related control variables included several known risk factors for allergies and asthma, including BMI; the weekly amount of aerobic physical activity completed; the academic semester in which the survey was completed; and the use of alcohol, tobacco/E-cigarettes, cannabis, and other drugs. A more detailed description of variable definitions can be found in Table A.1 (available online).

### Statistical Analysis

The demographics and health-related characteristics of individuals by opioid misuse were described using frequencies (*n*) and percentages (%) for categorical factors. The chi-square test for independence was used to test for an association between categorical factors and opioid misuse. Twelve separate multivariable logistic regression models were used to answer the primary research question, namely, whether there is an association between opioid misuse and asthma, environmental, food, and pet/animal allergies. An interaction term between opioid misuse and race/ethnicity, gender identity, and age was included to test for effect measure modification. All models were adjusted for the confounding effects of demographic, other substance use, and health behavior–related independent variables in Table 1. Linearity between age and the log-odds of the outcomes was assessed with LOESS (locally estimated scatterplot smoothing) plots. The nonlinearity of age with all outcomes was addressed by categorizing age into 5 categories. The Osius-Rojek test and the area under the receiver operating characteristic curve were used to assess goodness of fit. Unadjusted ORs and AORs, with corresponding 95% CIs, were reported. Ratios of AORs (RORs) and their corresponding 95% CIs were computed as post hoc comparisons to probe interaction effects.^41^

**Table 1:** Demographic, Health, and Health Behavior Characteristics of U.S. College Students: ACHA-NCHA IIIb 2019–2024

	Opioid misuse^a^			
Variable	No *n* (%^b^)	Yes *n* (%^b^)	*p*-value^c^	Total *n* (%^b^)
Total^d^	515,447 (96.2)	20,462 (3.8)		535,909 (100)
Independent variables				
Age, years			<0.001	
18–19	149,489 (29.3)	2,643 (13.1)		152,132 (28.7)
20–22	195,453 (38.4)	5,307 (26.4)		200,760 (37.9)
23–25	65,227 (12.8)	2,797 (13.9)		68,024 (12.8)
26–30	52,560 (10.3)	3,541 (17.6)		56,101 (10.6)
>30	46,767 (9.2)	5,831 (29.0)		52,598 (9.9)
Gender identity			<0.001	
Cisgender female	334,542 (65.1)	11,090 (54.7)		345,632 (64.7)
Cisgender male	151,155 (29.4)	7,427 (36.6)		158,582 (29.7)
Gender diverse	12,009 (2.3)	793 (3.9)		12,802 (2.4)
Transgender	15,831 (3.1)	976 (4.8)		16,807 (3.1)
Race/ethnicity			<0.001	
NH White	280,627 (54.4)	12,861 (62.9)		293,488 (54.8)
Hispanic or Latino/a/x	79,723 (15.5)	2,799 (13.7)		82,522 (15.4)
NH Asian or Asian American	76,165 (14.8)	1,090 (5.3)		77,255 (14.4)
NH biracial or multiracial	27,633 (5.4)	1,327 (6.5)		28,960 (5.4)
NH Black or African American	25,618 (5.0)	770 (3.8)		26,388 (4.9)
NH other/missing	14,905 (2.9)	699 (3.4)		15,604 (2.9)
American Indian or Native Alaskan	10,776 (2.1)	916 (4.5)		11,692 (2.2)
Alcohol use^e^			<0.001	
No	152,442 (29.6)	1,134 (5.6)		153,576 (28.7)
Yes	361,922 (70.4)	19,268 (94.4)		381,190 (71.3)
Tobacco or E-cigarette use^e^			<0.001	
No	358,480 (69.6)	3,661 (17.9)		362,141 (67.6)
Yes	156,551 (30.4)	16,769 (82.1)		173,320 (32.4)
Cannabis use^e^			<0.001	
No	313,369 (60.9)	2,348 (11.5)		315,717 (59.0)
Yes	201,048 (39.1)	18,056 (88.5)		219,104 (41.0)
Other drug use^f^			<0.001	
No	440,958 (85.5)	2,889 (14.1)		443,847 (82.8)
Yes	74,486 (14.5)	17,557 (85.9)		92,043 (17.2)
BMI			<0.001	
Underweight	26,557 (5.3)	855 (4.3)		27,412 (5.3)
Healthy weight	277,016 (55.4)	9,234 (46.6)		286,250 (55.0)
Overweight	115,955 (23.2)	5,245 (26.5)		121,200 (23.3)
Obese Class I	47,211 (9.4)	2,475 (12.5)		49,686 (9.6)
Obese Classes II + III	33,490 (6.7)	2,012 (10.2)		35,502 (6.8)
Exercise^g^			<0.001	
No	157,265 (30.9)	6,567 (32.5)		163,832 (31.0)
Yes	351,211 (69.1)	13,646 (67.5)		364,857 (69.0)
Semester			<0.001	
Fall 2019	36,780 (7.1)	1,676 (8.2)		38,456 (7.2)
Spring 2020	47,750 (9.3)	2,227 (10.9)		49,977 (9.3)
Fall 2020	12,804 (2.5)	475 (2.3)		13,279 (2.5)
Spring 2021	91,325 (17.7)	4,313 (21.1)		95,638 (17.8)
Fall 2021	31,149 (6.0)	1,038 (5.1)		32,187 (6.0)
Spring 2022	66,067 (12.8)	2,536 (12.4)		68,603 (12.8)
Fall 2022	32,379 (6.3)	1,121 (5.5)		33,500 (6.3)
Spring 2023	74,436 (14.4)	2,945 (14.4)		77,381 (14.4)
Fall 2023	23,578 (4.6)	636 (3.1)		24,214 (4.5)
Spring 2024	99,179 (19.2)	3,495 (17.1)		102,674 (19.2)
**Dependent variables**				
Asthma^h^			<0.001	
No	427,424 (84.0)	15,765 (78.5)		443,189 (83.7)
Yes	81,714 (16.0)	4,310 (21.5)		86,024 (16.3)
Environmental allergies^h^			<0.001	
No	358,981 (70.4)	13,158 (65.4)		372,139 (70.2)
Yes	150,658 (29.6)	6,952 (34.6)		157,610 (29.8)
Food allergies^h^			<0.001	
No	446,005 (87.6)	17,124 (85.2)		463,129 (87.5)
Yes	63,273 (12.4)	2,968 (14.8)		66,241 (12.5)
Pet/animal allergies^h^			<0.001	
No	439,564 (86.5)	16,599 (82.9)		456,163 (86.3)
Yes	68,837 (13.5)	3,436 (17.1)		72,273 (13.7)

a Opioid misuse includes ever use of illicit opioids or ever misuse of prescription opioids.

b Column percentage.

c Chi-square test for independence.

d Indicates total percentage of sample that did and did not misuse opioids.

e Ever used.

f Ever used cocaine, prescription stimulants, methamphetamine, inhalants, sedatives or sleeping pills, hallucinogens, or other drugs.

g Met U.S. recommended aerobic physical activity guidelines.

h Ever diagnosed. ACHA, American College Health Association; NCHA, National College Health Assessment; NH, non-Hispanic.

For the primary analysis, missing data were handled by listwise deletion, which assumes that missing data are missing completely at random. Among the 535,909 students with nonmissing opioid misuse data included in the analytic sample, 29,619 (5.5%) had missing data for at least 1 covariate, factor, or outcome. A multiple-imputation sensitivity analysis was conducted to assess the impact of possible violations of the missing completely at random assumption. Owing to the large sample size (*n*=541,093), 3 complete data sets, including exposure, outcomes, and covariates/factors, were multiply imputed using fully conditional specification. The relative efficiencies of estimates from 3 imputations are 85.71% and 90.91% when the fraction of missing information is 50% and 30%, respectively.^42^ Binary variables were imputed with predicted mean matching, and polychotomous variables were imputed with ordinal or generalized logistic regression. All exposures, outcomes (and their interactions with passive imputation), and covariates/factors were included as predictors for imputation as well as the following auxiliary variables: impediments to academic performance due to allergies, stress, and depression and self-reported diagnosis of attention-deficit disorder/attention-deficit/hyperactivity disorder, alcohol or other drug addiction, celiac disease, chronic pain, depression, anxiety, eating disorder, hepatitis B or C, and HIV or AIDS. Multiply imputed parameter estimates were pooled using Rubin’s rules, and the multivariate Wald test was used to test for interaction. All hypothesis tests were 2 sided and used a 5% significance level, and all statistical analyses were performed using SAS version 9.4 (SAS Institute, Inc., Cary, NC).

## RESULTS

Of 535,909 students, most were cisgender female (64.7%) and were aged 20–22 years (37.9%). The majority of students were NH White (54.8%), followed by Hispanic or Latino/a/x (15.4%), NH Asian or Asian American (14.4%), NH biracial or multiracial (5.4%), NH Black or African American (4.9%), NH other/missing (2.9%), and AI/NA (2.2%). Most students reported having ever used alcohol (71.3%), but only 41.0%, 32.4%, and 17.2% reported having ever used cannabis, tobacco or E-cigarettes, and other drugs, respectively. Most students had a healthy BMI (55.0%) and met the American College of Sports Medicine guidelines for weekly aerobic exercise (69.0%) (Table 1).

Only 20,462 (3.8%) students reported opioid misuse. NH White, NH biracial or multiracial, NH other/missing, and AI/NA students comprise 62.9%, 6.5%, 3.4%, and 4.5%, respectively, of the students who misuse opioids. In contrast, 54.4%, 5.4%, 2.9%, and 2.1% of the students who do not misuse opioids were NH White, NH biracial or multiracial, NH other/missing, and AI/NA, respectively (*p*<0.001) (Table 1). Similarly, the gender identity distribution shifted more toward students who are cisgender male (36.6% vs 29.4%), gender diverse (3.9% vs 2.3%), and transgender (4.8% vs 3.1%) among students who reported opioid misuse than among those who did not report misuse (*p*<0.001) (Table 1). Finally, students who reported opioid misuse tended to be older, with 29.0% being aged >30 years and only 13.1% being aged 18–19 years (*p*<0.001) (Table 1).

Opioid misuse is positively associated with asthma, because 21.5% of students reporting opioid misuse also reported ever being diagnosed with asthma, compared with 16.0% of students who did not report opioid misuse (*p*<0.001) (Table 1). Similarly, the prevalence of environmental, food, and pet/animal allergies is 5, 2.4, and 3.6 percentage points higher, respectively, among students who reported opioid misuse than among those who did not report misuse (*p*<0.001) (Table 1).

**Figure 1:**
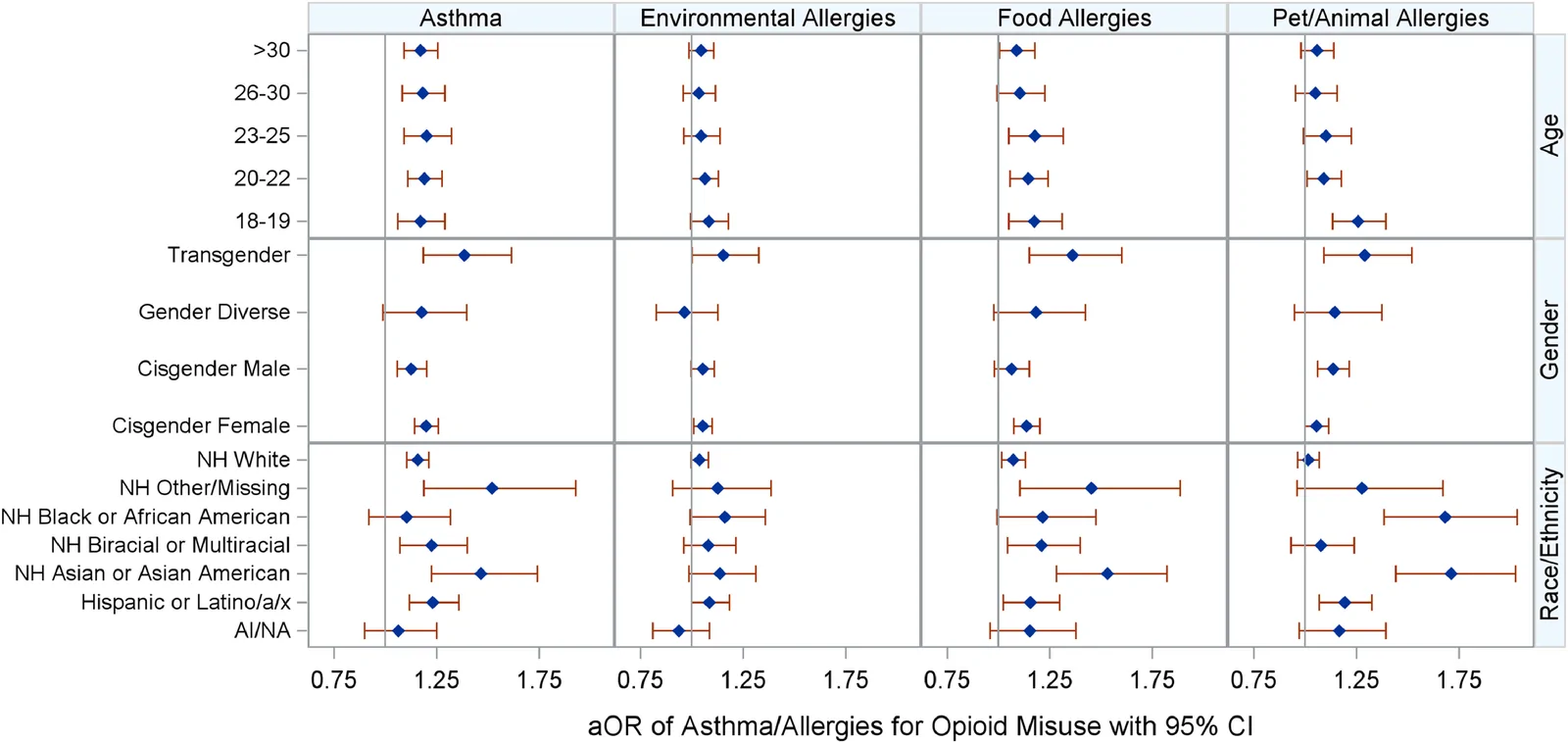
AORs and 95% CIs of each outcome for opioid misuse by levels of age, gender, or race/ethnicity. *Note*: Each cell in the panel represents AORs from logistic regression models for that outcome fit with an interaction term between opioid misuse and either age, gender, or race/ethnicity separately. Models are adjusted for all independent variables listed in Table 1. Reference line is drawn at OR=1. AI/NA, American Indian/Native Alaskan; NH, non-Hispanic.

Race/ethnicity was found to modify the association between opioid misuse and asthma (χ^2^[6]=13.72; *p*=0.0329), food allergies (χ^2^[6]=19.65; *p*=0.0032), and pet/animal allergies (χ^2^[6]=55.89; *p*<0.0001). Age and gender identity were not found to be significant effect modifiers of the association between opioid misuse and asthma, environmental allergies, food allergies, and pet/animal allergies (Figure 1 and Table 2). This pattern of effect modification is similar when missing data are multiply imputed, with gender identity having significant interaction effects for food and pet/animal allergies (Table A.2, available online). Figure 1 and Table 3 show that NH Asian or Asian American students had the strongest associations for food and pet/animal allergies, whereas NH other/missing students had the highest asthma AOR. The AORs of asthma and allergies for opioid misuse among NH Asian or Asian American students are 1.47 (95% CI=1.23, 1.74), 1.53 (95% CI=1.28, 1.82), and 1.71 (95% CI=1.44, 2.03) for asthma, food allergies, and pet/animal allergies, respectively. In contrast, the AORs for opioid misuse among NH White students are 1.16 (95% CI=1.10, 1.21), 1.07 (95% CI=1.02, 1.13), and 1.02 (95% CI=0.96, 1.07) for asthma, food allergies, and pet/animal allergies, respectively (Figure 1 and Table 3). The AORs of asthma and allergies for opioid misuse across race/ethnicity, gender, and age are similar when missing data are multiply imputed (Figure A.1, available online, and Table A.3, available online).

**Table 2:** Likelihood Ratio Joint Tests for Interaction With Opioid Misuse

Interaction term	χ^2^(df) *p*-value			
	Asthma	Environmental allergies	Food allergies	Pet/animal allergies
Opioid misuse × age	0.27 (4) 0.9919	0.81 (4) 0.9377	2.15 (4) 0.7078	8.50 (4) 0.0749^a^
Opioid misuse × gender	6.64 (3) 0.0843^a^	2.86 (3) 0.4137	7.40 (3) 0.0601^a^	7.07 (3) 0.0697^a^
Opioid misuse × race/ethnicity	**13.72 (6)** **0.0329**	6.70 (6) 0.3494	**19.65 (6)** **0.0032**	**55.89 (6)** **<0.0001**

*Note*: Boldface indicates statistical significance at the 5% significance level.

Each cell represents interaction term test statistic (degrees of freedom) and *p*-values from logistic regression models for the specified outcome fit with an interaction term between opioid misuse and either age, gender, or race/ethnicity separately. Models are adjusted for all independent variables listed in Table 1.

a Effects on the boundary of significance, 0.05<*p*<0.10.

**Table 3:** uORs and AORs of Each Outcome for Opioid Misuse by Race/Ethnicity

Race/ethnicity	Asthma		Environmental allergies		Food allergies		Pet/animal allergies	
	uOR (95% CI)	AOR (95% CI)	uOR (95% CI)	AOR (95% CI)	uOR (95% CI)	AOR (95% CI)	uOR (95% CI)	AOR (95% CI)
NH White	**1.31 (1.25, 1.36)**	**1.16 (1.10, 1.21)**	**1.18 (1.14, 1.22)**	1.04 (1.00, 1.08)^a^	**1.11 (1.06, 1.17)**	**1.07 (1.02, 1.13)**	**1.15 (1.09, 1.20)**	1.02 (0.96, 1.07)
Hispanic or Latino/a/x	**1.39 (1.27, 1.53)**	**1.23 (1.12, 1.36)**	**1.23 (1.14, 1.34)**	1.09 (1.00, 1.18)^a^	**1.18 (1.05, 1.33)**	**1.15 (1.02, 1.30)**	**1.34 (1.21, 1.49)**	**1.19 (1.07, 1.33)**
NH Asian or Asian American	**1.71 (1.45, 2.02)**	**1.47 (1.23, 1.74)**	**1.21 (1.06, 1.39)**	1.14 (0.98, 1.31)^a^	**1.58 (1.34, 1.87)**	**1.53 (1.28, 1.82)**	**1.99 (1.69, 2.34)**	**1.71 (1.44, 2.03)**
NH biracial or multiracial	**1.39 (1.23, 1.58)**	**1.23 (1.07, 1.40)**	**1.21 (1.08, 1.36)**	1.08 (0.96, 1.22)	**1.29 (1.12, 1.49)**	**1.21 (1.04, 1.40)**	**1.26 (1.10, 1.45)**	1.08 (0.93, 1.24)
NH Black or African American	**1.30 (1.10, 1.55)**	1.10 (0.92, 1.32)	**1.29 (1.11, 1.51)**	1.16 (0.99, 1.36)^a^	**1.31 (1.08, 1.58)**	1.21 (0.99, 1.47)^a^	**1.94 (1.61, 2.33)**	**1.68 (1.38, 2.04)**
NH other/missing	**1.97 (1.59, 2.44)**	**1.52 (1.19, 1.93)**	**1.37 (1.13, 1.66)**	1.13 (0.91, 1.39)	**1.67 (1.32, 2.12)**	**1.45 (1.10, 1.89)**	**1.63 (1.27, 2.08)**	1.28 (0.96, 1.67)^a^
AI/NA	**1.24 (1.06, 1.45)**	1.06 (0.90, 1.25)	1.09 (0.95, 1.26)	0.94 (0.81, 1.09)	**1.29 (1.09, 1.53)**	1.15 (0.96, 1.38)	**1.39 (1.17, 1.66)**	1.17 (0.97, 1.39)^a^

*Note*: Boldface indicates statistical significance at the 5% significance level.

AORs are estimated from logistic regression models for each outcome fit with an interaction term between opioid misuse and race/ethnicity separately. AORs are adjusted for all independent variables listed in Table 1.

a Effects on the boundary of significance, 0.05<*p*<0.10. AI/NA, American Indian or Native Alaskan; NH, non-Hispanic; uOR, unadjusted OR.

The ratio of AORs confirms this, because the AOR of asthma for opioid misuse in NH Asian or Asian American students is 1.28 times greater than the AOR in NH White students (ROR=1.28; 95% CI=1.06, 1.52) (Table A.4, available online). Similarly, the AOR in NH Asian or Asian American students is 1.43 times greater (ROR=1.43; 95% CI=1.19, 1.71) and 1.69 times greater (ROR=1.69; 95% CI=1.42, 2.01) than the AOR of food allergies for opioid misuse and the AOR of pet/animal allergies for opioid misuse in NH White students, respectively (Tables A.5 and A.6, available online). In general, the strength of the association between opioid misuse and asthma/allergies tends to be larger in NH Asian or Asian American students than in NH White, Hispanic or Latino/a/x, NH biracial or multiracial, and AI/NA students (Tables A.4–6, available online).

The association between opioid misuse and asthma (AOR=1.52; 95% CI=1.19, 1.93), food allergies (AOR=1.45; 95% CI=1.10, 1.89), and pet/animal allergies (AOR=1.28; 95% CI=0.96, 1.67) was also strong among students with NH other/missing identities (Figure 1 and Table 3). In fact, the association in NH other/missing students was 1.31 times larger for asthma (ROR=1.31; 95% CI=1.03, 1.68) and 1.35 times larger for food allergies (ROR=1.35; 95% CI=1.03, 1.78) than in NH White students (Tables A.4 and A.5, available online). NH Black or African American students had the second-largest association between opioid misuse and pet/animal allergies (AOR=1.68; 95% CI=1.38, 2.04) (Table 3), and it was larger than the association in NH White (ROR=1.66; 95% CI=1.36, 2.02), Hispanic or Latino/a/x (ROR=1.41; 95% CI=1.13, 1.76), NH biracial or multiracial (ROR=1.56; 95% CI=1.23, 1.98), and AI/NA (ROR=1.45; 95% CI=1.11, 1.89) students (Table A.6, available online).

## DISCUSSION

This cross-sectional study in a national sample of college students found that the prevalence of self-reported diagnoses of asthma, environmental allergies, food allergies, and pet/animal allergies was higher among students who reported ever misusing opioids than among those who did not report misusing opioids. Moreover, race/ethnicity modified the association between opioid misuse and asthma, food allergies, and pet/animal allergies but not environmental allergies. Opioid misuse was generally associated with higher odds of asthma and allergic outcomes, with particularly strong associations for asthma, food allergies, and pet/animal allergies in selected racial/ethnic groups. Specifically, NH Asian or Asian American students had particularly elevated associations for asthma, food allergies, and pet/animal allergies; NH other/missing students had the highest asthma AOR; and NH Black or African American students had one of the strongest associations for pet/animal allergies.

The findings are consistent with hypotheses that opioids may influence immune and inflammatory pathways relevant to asthma and allergic disease, including immunomodulatory effects and mast cell–mediated histamine release^26^^,^^27^^,^^29^; however, the cross-sectional design precludes causal inference, and temporality remains unclear. In addition, the epidemiologic literature has mixed findings on these associations. One Australian registry-based cohort study and one Swedish registry-based cohort study found that prenatal opioid exposure was associated with an increased risk of asthma in early childhood after multivariable adjustment for measured confounders.^43^^,^^44^ However, Shaheen et al.^44^ also conducted a sibling-matched case-control sensitivity analysis and found that prenatal opioid exposure was not associated with asthma in early childhood, which suggests that unmeasured maternal-specific confounders may explain their findings. Similarly, another Scandinavian registry-based cohort study found no association between long-term prenatal opioid exposure and early childhood asthma.^45^

Among adults who use illicit opioids, a meta-analysis found the prevalence of asthma to be 8.5% (95% prediction interval=0.2%, 74.0%) and 20.2% (95% prediction interval=4.2%, 59.2%) among those who inject illicit opioids and those who inhale illicit opioids, respectively, which were both noted to be higher than the general population prevalence of asthma.^46^ Among the broader population of U.S. adults, a retrospective cohort study utilizing a large national database of electronic health records found that patients with asthma were 2.12 times more likely to be diagnosed with opioid use disorder than patients without asthma.^22^ These results extend prior literature by demonstrating associations between opioid exposure and asthma, food allergies, and pet/animal allergies in a young, likely healthier sample of U.S. college students.

A novel finding from this study is that race/ethnicity modifies the association between opioid misuse and asthma, food allergies, and pet/animal allergies, with stronger associations observed in selected racial/ethnic minority groups. One hypothesis for the observed effect modification is that opioid misuse may interact with social and environmental exposures that are unevenly distributed across race/ethnicity and are established risk factors for asthma and allergic diseases. Prior studies have associated race/ethnicity with differences in indoor allergen exposure patterns, including exposure to multiple indoor allergens and variation in the types of indoor allergens encountered, as well as with allergic sensitization, including total/specific IgE and sensitization to at least 1 allergen.^47^, ^48^, ^49^ Racial and ethnic minority groups may also experience disproportionate exposure to environmental risk factors for asthma and allergies, including air pollution, household pests and mold, and environmental tobacco smoke.^15^^,^^50^, ^51^, ^52^ Notably, many prior studies tend to focus on Hispanics, NH Whites, and NH Black or African Americans, whereas this study found particularly strong associations among NH Asian or Asian American students for food and pet/animal allergies, among NH other/missing students for asthma, and among NH Black or African American students for pet/animal allergies. Thus, future research should include racial and ethnic identities not typically considered in health research.

In the context of the cross-sectional design of this study, the effect modification by race/ethnicity should be interpreted as the correlation or association between opioid misuse and asthma/allergies being larger in certain racial/ethnic minority college students and not as evidence of inherent biological differences. Effect modification by race/ethnicity should be interpreted in light of the structural determinants that pattern exposure to asthma/allergy triggers (e.g., housing quality, pollution, psychosocial stress, access to preventive care) and substance use risk environments. Because the ACHA-NCHA IIIb data set lacks direct measures of residential segregation and neighborhood context, race/ethnicity may partially proxy these conditions; future studies should incorporate multilevel measures of neighborhood and structural exposures to clarify the mechanisms underlying heterogeneous associations.^36^, ^37^, ^38^

### Limitations

This study is subject to several limitations. First, this study used a cross-sectional design, and thus no conclusions on causation or temporality between opioid misuse and asthma/allergies can be made from these results. This relationship is likely bidirectional, such that individuals with asthma or allergic disease may be more likely to receive opioid prescriptions for pain (or have comorbid mental health conditions associated with misuse).^22^ There may also be residual confounding bias due to unmeasured shared social/behavioral risk factors for opioid misuse and asthma/allergies. Second, generalizability may be limited owing to students self-selecting into the survey and low response rates. However, prior studies show that substance use prevalence estimates from the NCHA are similar, regardless of whether sampling weights are used to correct for demographic imbalances,^53^ and that NCHA substance use prevalence estimates are similar to estimates in young adult college students from the National Survey on Drug Use and Health and the Monitoring the Future survey.^54^ Third, the opioid misuse measure, on the basis of any lifetime heroin use or nonmedical prescription opioid use, was broad. Future studies that use measures of opioid exposure considering the type of opioids used, route of use, frequency, and dose of use would further clarify the association between opioid misuse and asthma/allergies. Finally, indications of asthma and allergies are self-reported and not confirmed by spirometry or skin prick tests.

## CONCLUSIONS

This study adds further evidence to the emerging link between opioid use and asthma and allergic diseases and highlights the need for further basic and translational research. Opioids are a broad class of drugs, and opioid misuse is a broad term for a variety of consumption behaviors. More epidemiologic research on whether specific types of opioids, routes of use, and the frequency and dose of opioid use affect the development and severity of asthma and allergies is needed. At the same time, continued basic research on how the immune system simultaneously interacts with opioids, allergens, and environmental exposures is needed to develop biopsychosocial conceptual models that can be used to guide the prevention and harm reduction of both opioid addiction and asthma and allergic diseases.

## CRediT authorship contribution statement

**Fares Qeadan:** Conceptualization, Methodology, Validation, Supervision, Project administration, Writing – review & editing. **Stephane Beaudin:** Data curation, Visualization, Writing – review & editing. **Aisha Arshad:** Investigation, Writing – original draft. **William A. Barbeau:** Formal analysis, Writing – original draft, Writing – review & editing.

## Appendix Supplementary materials

Image, application 1

## Footnotes

Supplementary material associated with this article can be found in the online version at doi:10.1016/j.focus.2026.100525.

## References

[1] Understanding the opioid overdose epidemic. Centers for Disease Control and Prevention. https://www.cdc.gov/overdose-prevention/about/understanding-the-opioid-overdose-epidemic.html. Updated 2025. Accessed March 2, 2026.

[2] Chandler R.K., Villani J., Clarke T., McCance-Katz E.F., Volkow N.D. (2020). Addressing opioid overdose deaths: the vision for the HEALing communities study. Drug Alcohol Depend.

[3] Dowell D., Ragan K.R., Jones C.M., Baldwin G.T., Chou R. (2022). CDC clinical practice guideline for prescribing opioids for pain - United States, 2022. MMWR Recomm Rep.

[4] Substance Abuse and Mental Health Services Administration, Key substance use and mental health indicators in the United States: results from the 2021 National Survey on Drug Use and Health (HHS Publication No. PEP22-07-01-005, NSDUH Series H-57), 2022https://www.samhsa.gov/data/report/2021-nsduh-annual-national-report. Accessed September 4, 2026.

[5] Substance Abuse and Mental Health Services Administration, Results from the 2021 national survey on drug use and health: detailed tables, 2023.https://www.samhsa.gov/data/report/2021-nsduh-detailed-tables. Accessed September 4, 2026.

[6] Substance use among youth. Centers for Disease Control and Prevention.https://www.cdc.gov/youth-behavior/risk-behaviors/substance-use-among-youth.html?CDC_AAref_Val=https://www.cdc.gov/healthyyouth/substance-use/index.htm. Updated 2024. Accessed August 28, 2025.

[7] Fishman M., Wenzel K., Scodes J. (2020). Young adults have worse outcomes than older adults: secondary analysis of a medication trial for opioid use disorder. J Adolesc Health.

[8] Schulenberg J.E., Patrick M.E., Johnston L.D., O’Malley P.M., Bachman J.G., Miech R.A. (Published July 2021). https://eric.ed.gov/?id=ED615085.

[9] Garnett M.F., Miniño A.M. (2024). Drug overdose deaths in the United States, 2003–2023. NCHS Data Brief.

[10] Qeadan F., Azagba S., Barbeau W.A. (2022). Associations between discrimination and substance use among college students in the United States from 2015 to 2019. Addict Behav.

[11] Qeadan F., Madden E.F., Bern R. (2021). Associations between opioid misuse and social relationship factors among American Indian, Alaska Native, and Native Hawaiian college students in the U.S. Drug Alcohol Depend.

[12] Most recent asthma data. Centers for Disease Control and Prevention. https://www.cdc.gov/asthma-data/about/most-recent-asthma-data.html. Updated 2024. Accessed July 14, 2025.

[13] Ng A.E., Boersma P. (2023). Diagnosed allergic conditions in adults: United States, 2021. NCHS Data Brief.

[14] American College Health Association (Published 2022). https://www.acha.org/wp-content/uploads/2024/07/NCHA-III_FALL_2021_REFERENCE_GROUP_EXECUTIVE_SUMMARY.pdf.

[15] Miller R.L., Grayson M.H., Strothman K. (2021). Advances in asthma: new understandings of asthma’s natural history, risk factors, underlying mechanisms, and clinical management. J Allergy Clin Immunol.

[16] Nurmagambetov T., Kuwahara R., Garbe P. (2018). The economic burden of asthma in the United States, 2008–2013. Ann Am Thorac Soc.

[17] Wang LY, Zhong Y, Wheeler L. Direct and indirect costs of asthma in school-age children. *Prev Chronic Dis*. 2005;2(1):A11. http://www.cdc.gov/pcd/issues/2005/jan/04_0053.htm. Accessed July 22, 2026. PMC1323314 15670464

[18] Allergens and pollen. Centers for Disease Control and Prevention. https://www.cdc.gov/climate-health/php/effects/allergens-and-pollen.html. Updated 2024. Accessed Jun 24, 2024.

[19] Dharmage S.C., Perret J.L., Custovic A. (2019). Epidemiology of asthma in children and adults. Front Pediatr..

[20] Hossny E., Ebisawa M., El-Gamal Y. (2019). Challenges of managing food allergy in the developing world. World Allergy Organ J.

[21] Loh W., Tang M.L.K. (2018). The epidemiology of food allergy in the global context. Int J Environ Res Public Health.

[22] Qeadan F., McCunn A., Tingey B., Price R., Bobay K.L., Saeed A.I. (2024). Investigating the association between asthma and opioid use disorder with interactions of anxiety and depression among a national sample of the U.S. population. J Asthma.

[23] Liang X., Liu R., Chen C., Ji F., Li T. (2016). Opioid system modulates the immune function: a review. Transl Perioper Pain Med.

[24] Ogulur I., Pat Y., Ardicli O. (2021). Advances and highlights in biomarkers of allergic diseases. Allergy.

[25] Platts-Mills T.A. (2001). The role of immunoglobulin E in allergy and asthma. Am J Respir Crit Care Med.

[26] Sun Q., Li Z., Wang Z. (2023). Immunosuppression by opioids: mechanisms of action on innate and adaptive immunity. Biochem Pharmacol.

[27] Bettinger J.J., Friedman B.C. (2024). Opioids and immunosuppression: clinical evidence, mechanisms of action, and potential therapies. Palliat Med Rep.

[28] Barke K.E., Hough L.B. (1993). Opiates, mast cells and histamine release. Life Sci..

[29] Baldo B.A. (2023). MRGPRX. MRGPRX2, drug pseudoallergies, inflammatory diseases, mechanisms and distinguishing MRGPRX2- and IgE/FcεRI-mediated events. Br J Clin Pharmacol.

[30] Sutaria N., Adawi W., Goldberg R., Roh Y.S., Choi J., Kwatra S.G. (2022). Itch: pathogenesis and treatment. J Am Acad Dermatol.

[31] Malekzad F., Arbabi M., Mohtasham N. (2009). Efficacy of oral naltrexone on pruritus in atopic eczema: a double-blind, placebo-controlled study. J Eur Acad Dermatol Venereol.

[32] Frech T., Novak K., Revelo M.P. (2011). Low-dose naltrexone for pruritus in systemic sclerosis. Int J Rheumatol.

[33] Ekelem C., Juhasz M., Khera P., Mesinkovska N.A. (2019). Utility of naltrexone treatment for chronic inflammatory dermatologic conditions: a systematic review. JAMA Dermatol.

[34] Forno E., Celedon J.C. (2009). Asthma and ethnic minorities: socioeconomic status and beyond. Curr Opin Allergy Clin Immunol.

[35] Jones C.J., Paudyal P., West R.M. (2022). Burden of allergic disease among ethnic minority groups in high-income countries. Clin Exp Allergy.

[36] Martinez A., de la Rosa R., Mujahid M., Thakur N. (2021). Structural racism and its pathways to asthma and atopic dermatitis. J Allergy Clin Immunol.

[37] Alexander D., Currie J. (2017). Is it who you are or where you live? Residential segregation and racial gaps in childhood asthma. J Health Econ.

[38] Ryan P.H., Zanobetti A., Coull B.A. (2024). The legacy of redlining: increasing childhood asthma disparities through neighborhood poverty. Am J Respir Crit Care Med.

[39] Lederer A.M., Hoban M.T. (2022). The development of the American College Health Association-National College Health Assessment III: an improved tool to assess and enhance the health and well-being of college students. J Am Coll Health.

[40] All NCHA survey reports. American College Health Association. https://www.acha.org/ncha/data-results/survey-results/all-ncha-survey-reports/. Accessed March 3, 2025.

[41] Altman D.G., Bland J.M. (2003). Interaction revisited: the difference between two estimates. BMJ.

[42] The MI procedure - multiple imputation efficiency. SAS Institute Inc. https://documentation.sas.com/doc/en/pgmsascdc/9.4_3.5/statug/statug_mi_details54.htm. Updated 2025. Accessed Mar 13, 2026.

[43] Kelty E., Rae K., Jantzie L.L., Wyrwoll C.S., Preen D.B. (2024). Prenatal opioid exposure and immune-related conditions in children. JAMA Netw Open.

[44] Shaheen S.O., Lundholm C., Brew B.K., Almqvist C. (2019). Prescribed analgesics in pregnancy and risk of childhood asthma. Eur Respir J.

[45] Odsbu I., Handal M., Hjellvik V. (2023). Prenatal opioid exposure and risk of asthma in childhood: a population-based study from Denmark, Norway, and Sweden. Front Pharmacol.

[46] Hulin J., Brodie A., Stevens J., Mitchell C. (2020). Prevalence of respiratory conditions among people who use illicit opioids: a systematic review. Addiction.

[47] Salo P.M., Wilkerson J., Rose K.M. (2018). Bedroom allergen exposures in U.S. households. J Allergy Clin Immunol.

[48] Sitarik A., Havstad S., Kim H. (2020). Racial disparities in allergic outcomes persist to age 10 years in black and white children. Ann Allergy Asthma Immunol.

[49] Wegienka G., Havstad S., Joseph C.L. (2012). Racial disparities in allergic outcomes in African Americans emerge as early as age 2 years. Clin Exp Allergy.

[50] Mitchell E.A., Beasley R., Keil U., Montefort S., Odhiambo J., ISAAC Phase Three Study Group (2012). The association between tobacco and the risk of asthma, rhinoconjunctivitis and eczema in children and adolescents: analyses from Phase Three of the ISAAC programme. Thorax.

[51] Perry T.T., Grant T.L., Dantzer J.A., Udemgba C., Jefferson A.A. (2024). Impact of socioeconomic factors on allergic diseases. J Allergy Clin Immunol.

[52] Raghuveer G., White D.A., Hayman L.L. (2016). Cardiovascular consequences of childhood secondhand tobacco smoke exposure: prevailing evidence, burden, and racial and socioeconomic disparities: a scientific statement from the American Heart Association. Circulation.

[53] Kerr D.C.R., Bae H., Alley Z.M. (2021). Enhancing gender and ethnic representativeness of NCHA-II data with survey weights: the examples of substance use prevalence and state marijuana legalization. J Am Coll Health.

[54] Kerr D.C.R., Bae H. (2025). Comparing National College Health Assessment with other surveys of cannabis use and binge drinking among young adult college students 2008–2018. J Am Coll Health.

