# Supplementary material for: Opioid Misuse and Asthma and Allergic Diseases in College Students: The Role of Race and Ethnicity
Source: AJPM Focus. 2026 Jul 7;5(5):100525. doi: 10.1016/j.focus.2026.100525 (PMC13586716; doi:10.1016/j.focus.2026.100525)
Supplement: Supplementary file 1 [file mmc1.pdf]

Opioid Misuse and Asthma and Allergic Diseases in College Students: The Role of Race and Ethnicity

Fares Qeadan, PhD, MS, Stephane Beaudin, PhD, Aisha Arshad, BA, William A. Barbeau, MSTAT

AJPM Focus

Table A.1. Variable Construction

Variable	Survey Question	Possible Survey Responses	Categorization (if applicable)
Opioid Misuse	Preamble: In your life, which of the following substances have you ever used? For prescription medications, please report nonmedical use only. "Nonmedical use" means taking prescription drugs just for the feeling or experience they cause or taking them more often or at higher doses than prescribed.<br><br>Substance options:<br>• Prescription opioids (morphine, codeine, fentanyl, oxycodone [OxyContin, Percocet], hydrocodone [Vicodin], methadone, buprenorphine [Suboxone], etc.) [Please report nonmedical use only.]<br>• Heroin	No<br>Yes	Yes if students endorsed yes to either prescription opioids or heroin; otherwise no
Age	How old are you?	Numeric response	• 18-19<br>• 20-22<br>• 23-25<br>• 26-30<br>• >30
Gender	Three separate survey questions:<br>• N3Q67A: What sex were you assigned at birth?	• N3Q67A<br>○ Female<br>○ Male	• Anyone one who reported identifying as transgender (N2Q67B=Yes) or as a trans

	• N3Q67B: Do you identify as transgender?<br>• N3Q67C: Which term do you use to describe your gender identity?	○ Intersex<br>• N3Q67B<br>○ No<br>○ Yes<br>• N3Q67C<br>○ Woman or female<br>○ Man or male<br>○ Trans woman<br>○ Trans man<br>○ Genderqueer<br>○ Agender<br>○ Genderfluid<br>○ Intersex<br>○ Non-binary<br>○ My identity is not listed above	woman or trans man were categorized as transgender.<br>• Otherwise anyone who reported genderqueer, agender, genderfluid, intersex, non-binary, or other not listed identity were categorized as gender diverse<br>• Otherwise anyone whose sex at birth was male or female and identity was not concordant with their sex was categorized as transgender<br>• Otherwise anyone with concordant sex and gender identity or responded to either sex or gender identity but missing in the other was categorized as cisgender female or cisgender male based on their responses.
Race/Ethnicity	How do you usually describe yourself? (Please select ALL that apply)	American Indian or Native Alaskan<br>Asian or Asian American<br>Black or African American<br>Hispanic or Latino/a/x<br>Middle Eastern/North African (MENA) or Arab Origin<br>Native Hawaiian or Other Pacific Islander Native<br>White<br>Biracial or Multiracial<br>My identity is not listed above	(1) American Indian or Native Alaskan*<br>(2) NH Asian or Asian American<br>(3) NH Black or African American<br>(4) Hispanic or Latino/a/x**<br>(5) NH White<br>(6) NH Biracial or Multiracial***<br>(7) NH Other/Missing (Middle Eastern/North African (MENA) or Arab Origin, Native Hawaiian or Other Pacific Islander Native, My identity is not listed above or no selections of any options)<br><br>*Anyone who marked American Indian or Native Alaskan were categorized as such, regardless of other responses.<br><br>**Anyone who marked Hispanic or Latino/a/x and not American Indian or

			Native Alaskan were categorized as such, regardless of other responses.<br><br>***Those who report more than one race are also categorized as NH Biracial or Multiracial
Preamble for substance use questions: In your life, which of the following substances have you ever used? For prescription medications, please report nonmedical use only. "Nonmedical use" means taking prescription drugs just for the feeling or experience they cause or taking them more often or at higher doses than prescribed.			
Alcohol use	Alcoholic beverages (beer, wine, liquor, etc.)	No<br>Yes	
Tobacco or e-cigarette use	Tobacco or nicotine delivery products (cigarettes, e-cigarettes, Juul or other vape products, chewing tobacco, cigars, etc.)	No<br>Yes	
Cannabis use	Cannabis (marijuana, weed, hash, edibles, vaped cannabis, etc.) [Please report nonmedical use only.]	No<br>Yes	
Other drug use	• Cocaine (coke, crack, etc.)<br>• Prescription stimulants (Ritalin, Concerta, Dexedrine, Adderall, diet pills, etc.) [Please report nonmedical use only.]<br>• Methamphetamine (speed, crystal meth, ice, etc.)<br>• Inhalants (poppers, nitrous, glue, gas, paint thinner, etc.)<br>• Sedatives or Sleeping Pills (Valium, Ativan, Xanax, Klonopin, Librium, Rohypnol, GHB, etc.) [Please report nonmedical use only.]<br>• Hallucinogens (Ecstasy, MDMA, Molly, LSD, acid, mushrooms, PCP, Special K, etc.)<br>• Other – Specify:	No<br>Yes	If students endorsed yes to any substances listed, they were categorized as yes. Otherwise they were categorized as no.
BMI	• What is your height in feet (') and inches (")?	Numeric response	BMI was computed by ACHA and categorized as

	• What is your weight in pounds?		• BMI <18.5 Underweight<br>• BMI 18.5-24.9 Healthy Weight<br>• BMI 25-29.9 Overweight<br>• BMI 30-34.9 Class I Obesity<br>• BMI >35 Class II + III Obesity
Exercise	For the next two questions, the levels of physical activity intensity can be characterized in terms of breathing difficulty. A person doing moderate physical activity can typically talk, but not sing while doing the activity. A person doing vigorous physical activity typically cannot say more than a few words without pausing for a breath while doing the activity.<br><br>• In the last 7 days, how many (total) minutes did you spend doing moderate physical activity? Examples: brisk walking, dancing, or household chores.<br><br>• In the last 7 days, how many (total) minutes did you spend doing vigorous physical activity? Examples: running, swimming laps, or hiking.	Numeric response	Yes if student reported 150 or more minutes per week of moderate aerobic activity, where 1 minute of vigorous activity equals 2 minutes of moderate activity. Otherwise no.
Dependent Variables			
Asthma	Have you ever been diagnosed by a healthcare or mental health professional with any of the following ongoing or chronic conditions? [Asthma]	No<br>Yes	
Environmental Allergies	Have you ever been diagnosed by a healthcare or mental health professional with any of the following ongoing or chronic conditions? [Allergies – environmental (for example: pollen, grass, dust, mold)]	No<br>Yes	

Food Allergies	Have you ever been diagnosed by a healthcare or mental health professional with any of the following ongoing or chronic conditions? [Allergies – food allergy]	No<br>Yes	
Pet/Animal Allergies	Have you ever been diagnosed by a healthcare or mental health professional with any of the following ongoing or chronic conditions? [Allergies – animals/pets]	No<br>Yes	

Table A. 2. Multiple imputation sensitivity analysis, multivariate Wald tests for interaction. Each cell represents the joint interaction term F test statistic (numerator degrees of freedom, denominator degrees of freedom) and p-values from logistic regression models for the specified outcome fit with an interaction term between opioid misuse and either age, gender, or race/ethnicity separately. Models are adjusted for all independent variables listed in Table 1. Model parameters are pooled across 3 imputations (m=3). Bold indicates statistical significance at the 5% significance level. Italics indicates effects on the boundary of significance, 0.05<p-value<0.10.

	$F(df_1,df_2)$<br>p-value			
	Asthma	Environmental Allergies	Food Allergies	Pet/Animal Allergies
Opioid Misuse x Age	0.02 (4, 2,832.1)<br>0.999	0.10 (4, 2,519.7)<br>0.981	0.36 (4, 820.1)<br>0.837	1.91 (4, 72.0)<br>0.109
Opioid Misuse x Gender	*2.51 (3, 2,069.4)*<br>*0.057*	1.29 (3, 381.5)<br>0.279	**4.79 (3, 386.1)**<br>**0.003**	**4.80 (3, 170.7)**<br>**0.003**
Opioid Misuse x Race/Ethnicity	**4.28 (6, 5,084.3)**<br>**<.001**	1.18 (6, 330.7)<br>0.317	**4.74 (6, 4,579.4)**<br>**<.001**	**11.49 (6, 738.1)**<br>**<.001**

Figure A.1. Multiply imputed adjusted Odds Ratios (aOR) and 95% Confidence Intervals (CI) of each outcome for opioid misuse by levels of age, gender, or race/ethnicity. Each cell in the panel represents aORs from logistic regression models for that outcome fit with an interaction term between opioid misuse and either age, gender, or race/ethnicity separately. Models are adjusted for all independent variables listed in Table 1. Model parameters are pooled across 3 imputations (m=3). Reference line drawn at OR=1. NH=non-Hispanic. AI/NA= American Indian/Native Alaskan.

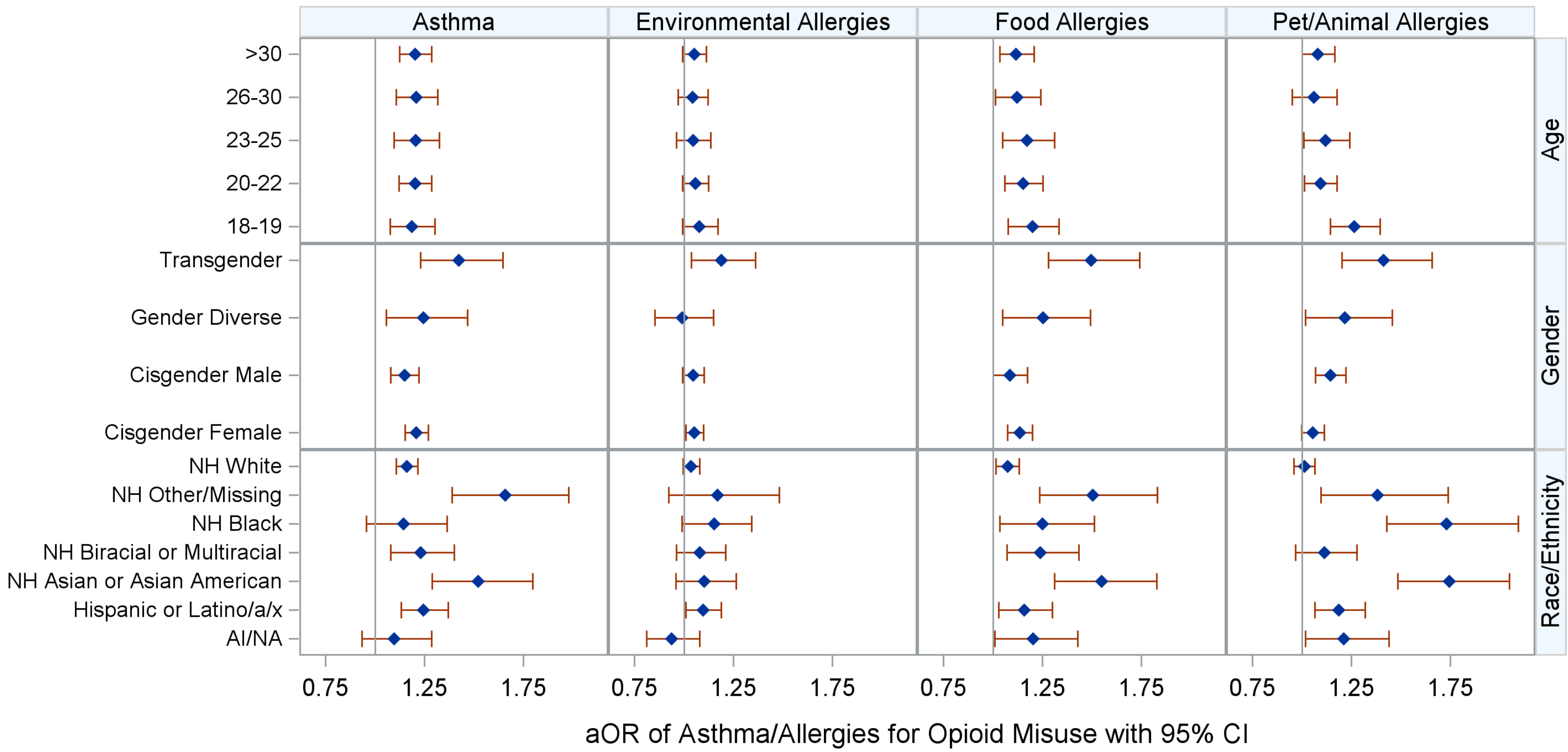

Table A.3. Multiply imputed Adjusted Odds Ratios (aOR) of each outcome for opioid misuse by race/ethnicity. aORs are estimated from logistic regression models for each outcome fit with an interaction term between opioid misuse and race/ethnicity separately. aORs are adjusted for all independent variables listed in Table 1. Model parameters are pooled across 3 imputations (m=3). Bold indicates statistical significance at the 5% significance level. Italics indicates effects on the boundary of significance, 0.05<p-value<0.10. NH=non-Hispanic. AI/NA=American Indian or Native Alaskan.

	Asthma	Environmental Allergies	Food Allergies	Pet/Animal Allergies
	aOR (95% CI)	aOR (95% CI)	aOR (95% CI)	aOR (95% CI)
NH White	**1.16 (1.11, 1.22)**	*1.04 (1.00, 1.08)*	**1.07 (1.02, 1.13)**	1.01 (0.96, 1.06)
Hispanic or Latino/a/x	**1.25 (1.13, 1.37)**	**1.10 (1.01, 1.19)**	**1.16 (1.03, 1.30)**	**1.18 (1.06, 1.32)**
NH Asian or Asian American	**1.52 (1.29, 1.80)**	1.10 (0.96, 1.26)	**1.55 (1.31, 1.83)**	**1.74 (1.48, 2.05)**
NH Biracial or Multiracial	**1.23 (1.08, 1.40)**	1.08 (0.96, 1.21)	**1.24 (1.07, 1.43)**	1.11 (0.97, 1.28)
NH Black	1.14 (0.96, 1.36)	*1.15 (0.99, 1.34)*	**1.25 (1.03, 1.51)**	**1.73 (1.43, 2.09)**
NH Other/Missing	**1.66 (1.39, 1.98)**	1.17 (0.92, 1.48)	**1.50 (1.24, 1.83)**	**1.38 (1.10, 1.74)**
AI/NA	1.10 (0.94, 1.29)	0.94 (0.81, 1.08)	**1.20 (1.01, 1.43)**	**1.21 (1.02, 1.44)**

Table A.4. Ratio of adjusted odds ratios (ROR) of asthma for opioid misuse with interaction by race/ethnicity. Rows indicate the reference group aOR. Bold indicates statistical significance at the 5% significance level. Italics indicates effects on the boundary of significance, 0.05<p-value<0.10. NH=non-Hispanic. AI/NA=American Indian or Native Alaskan.

	ROR (95% CI)<br>p-value						
	NH White	Hispanic or Latino/a/x	NH Asian	NH Biracial or Multiracial	NH Black	NH Other/Missing	AI/NA
NH White	Ref	1.06 (0.96-1.18)<br>0.267	**1.28 (1.06-1.52)<br>0.010**	1.06 (0.92-1.22)<br>0.423	0.95 (0.79-1.15)<br>0.617	**1.31 (1.03-1.68)<br>0.029**	0.92 (0.77-1.09)<br>0.323
Hispanic or Latino/a/x		Ref	*1.19 (0.98-1.45)<br>0.087*	0.99 (0.84-1.17)<br>0.946	0.90 (0.73-1.10)<br>0.291	1.23 (0.95-1.60)<br>0.111	0.862 (0.71-1.04)<br>0.126
NH Asian			Ref	0.84 (0.67-1.04)<br>0.107	**0.75 (0.59-0.97)<br>0.026**	1.04 (0.77-1.40)<br>0.810	**0.72 (0.57-0.92)<br>0.008**
NH Biracial or Multiracial				Ref	0.90 (0.72-1.13)<br>0.360	1.24 (0.94-1.63<br>0.122	0.87 (0.70-1.07)<br>0.184
NH Black					Ref	**1.38 (1.02-1.86)<br>0.036**	0.96 (0.75-1.23)<br>0.757
NH Other/Missing						Ref	**0.698 (0.522-0.934)<br>0.016**
AI/NA							Ref

Table A.5. Ratio of adjusted odds ratios (ROR) of food allergies for opioid misuse with interaction by race/ethnicity. Rows indicate the reference group aOR. Bold indicates statistical significance at the 5% significance level. Italics indicates effects on the boundary of significance, 0.05<p-value<0.10. NH=non-Hispanic. AI/NA=American Indian or Native Alaskan.

	ROR (95% CI)<br>p-value						
	NH White	Hispanic or Latino/a/x	NH Asian	NH Biracial or Multiracial	NH Black	NH Other/Missing	AI/NA
NH White	Ref	1.08 (0.95-1.23)<br>0.265	**1.43 (1.19-1.71)<br><0.001**	1.13 (0.97-1.32)<br>0.124	1.13 (0.92-1.39)<br>0.229	**1.35 (1.03-1.78)<br>0.029**	1.07 (0.89-1.29)<br>0.456
Hispanic or Latino/a/x		Ref	**1.33 (1.08-1.64)<br>0.009**	1.05 (0.87-1.26)<br>0.621	1.05 (0.84-1.32)<br>0.663	1.26 (0.94-1.69)<br>0.123	1.00 (0.80-1.24)<br>0.987
NH Asian			Ref	**0.79 (0.63-0.99)<br>0.042**	*0.79 (0.61-1.03)<br>0.083*	0.95 (0.69-1.30)<br>0.744	**0.75 (0.58-0.97)<br>0.026**
NH Biracial or Multiracial				Ref	1.00 (0.79-1.28)<br>0.977	1.20 (0.89-1.63)<br>0.239	0.95 (0.75-1.20)<br>0.678
NH Black					Ref	1.20 (0.86-1.67)<br>0.290	0.95 (0.73-1.24)<br>0.698
NH Other/Missing						Ref	0.79 (0.57-1.10)<br>0.159
AI/NA							Ref

Table A.6. Ratio of adjusted odds ratios (ROR) of pet/animal allergies for opioid misuse with interaction by race/ethnicity. Rows indicate the reference group aOR. Bold indicates statistical significance at the 5% significance level. Italics indicates effects on the boundary of significance, 0.05<p-value<0.10. NH=non-Hispanic. AI/NA=American Indian or Native Alaskan.

	ROR (95% CI)<br>p-value						
	NH White	Hispanic or Latino/a/x	NH Asian	NH Biracial or Multiracial	NH Black	NH Other/Missing	AI/NA
NH White	Ref	**1.18 (1.05-1.32)<br>0.007**	**1.69 (1.42-2.01)<br><0.001**	1.06 (0.91-1.23)<br>0.439	**1.66 (1.36-2.02)<br><0.001**	1.26 (0.95-1.67)<br>0.108	1.15 (0.95-1.38)<br>0.141
Hispanic or Latino/a/x		Ref	**1.44 (1.18-1.76)<br><0.001**	0.90 (0.76-1.08)<br>0.254	**1.41 (1.13-1.76)<br>0.002**	1.07 (0.80-1.44)<br>0.649	0.98 (0.79-1.20)<br>0.836
NH Asian			Ref	**0.63 (0.50-0.78)<br><0.001**	0.98 (0.76-1.27)<br>0.893	*0.75 (0.54-1.03)<br>0.076*	**0.68 (0.53-0.87)<br>0.002**
NH Biracial or Multiracial				Ref	**1.56 (1.23-1.98)<br><0.001**	1.19 (0.87-1.62)<br>0.279	1.08 (0.86-1.36)<br>0.488
NH Black					Ref	0.76 (0.54-1.06)<br>0.108	**0.69 (0.53-0.90)<br>0.006**
NH Other/Missing						Ref	0.91 (0.66-1.27)<br>0.589
AI/NA							Ref
